# An Unusual Increase in the CD38 Marker Observed in a Multiple Myeloma Patient With t(11;14) Translocation: A Case Report

**DOI:** 10.7759/cureus.63563

**Published:** 2024-07-01

**Authors:** Felix Rivera Troia, Fernando J Ocasio Villa

**Affiliations:** 1 Surgery, University of Medicine & Health Science, Mayaguez, PRI; 2 Genetics, Ponce Health Sciences University, Ponce, PRI; 3 Genetics, Western Oncology Cancer Center/Mayaguez Medical Center, Mayaguez, PRI

**Keywords:** immuo-oncology, malignant hematology, translocation 11 14, cd38, diagnosis of multiple myeloma

## Abstract

Multiple myeloma (MM) is one of the world's most recognized bone marrow (BM) cancers. It is considered a plasma cell dyscrasia in which normal plasma cells transform into malignant cells that produce large quantities of an abnormal immunoglobulin called monoclonal protein better known as M protein. This, in turn, is responsible for many of its bone and kidney-related manifestations. Many translocations are associated with the disease, such as t(11;14), t(4;14), and t(14;16). Of these, the most common is t(11;14). In this subset of MM, there is a specific genetic alteration affecting the CCND1 gene. Typically inactive in plasma cells, this gene, when disrupted, promotes uncontrolled cell proliferation. Simultaneously, there is a reduction in CD38 levels, a protein typically elevated in MM patients. This combination of genetic and protein expression is a defining feature of this subgroup within the MM spectrum.

In this report, we present a case of a 75-year-old male who was referred by an oncologist for comprehensive diagnostic testing. He was found to have significant hyperploidy involving trisomy 9 and an extra copy of CCND1 with concomitant trisomy 11q confirming a t(11;14) translocation. Further workup involving cytology revealed that the patient also expressed elevated levels of CD38, which, given this mutation, would be expected to be low in this patient population. We aim to highlight the importance and prognostic value of this mutation and further add to the already growing body of literature associated with this disease.

## Introduction

Multiple myeloma (MM) is a plasma cell dyscrasia that accounts for approximately 10% of all hematologic-associated malignancies [1]. It is caused by the uncontrolled proliferation of monoclonal plasma cells in the bone marrow (BM). These cells, in turn, produce large quantities of an abnormal immunoglobulin known as monoclonal protein or M protein, causing damage to the kidneys, lytic bone lesions, anemia, and hypercalcemia [2]. The disease affects one out of every 103 men and one out of every 131 women, making it more common in males than in females. Some risk factors associated with the disease include but are not limited to a family history of MM, excess body weight, African American ethnicity, which increases the risk of MM by twofold, and older age (median age of onset: around 65 years) [3].

The exact etiology of MM is not well-understood as yet; however, progress has been made in understanding certain changes in DNA that make plasma cells become cancerous. For example, deletions in certain genes like chromosome 17p are associated with the loss of TP53, an important tumor suppressor protein. This protein functions as a proofreading mechanism during the cell cycle, modifying errors when spotted or inducing apoptosis in cells when unfixable mutations are encountered [4]. Therefore, a mutation in this gene will lead to an absence of proofreading and a continuous proliferation of mutated cells. Mutations in this gene are observed in approximately 10% of MM patients and are associated with a poor prognosis [5].

Our focus in this case report is on the t(11;14) mutation, a translocation that implicates CCND1 and immunoglobulin heavy locus (IgH) genes. These mutations are found in approximately 15-20% of MM cases and are associated with a favorable prognosis. Patients with this mutation exhibit a unique biological feature compared to other MM cell types, characterized by an increase in the presence of the B-cell lineage membrane protein CD20, higher levels of the B-cell receptor CD79a, and a decrease in the expression of both CD38 and CD56 [6,7].

## Case presentation

The patient was a 75-year-old male with a past medical history of diabetes mellitus, osteoporosis, and benign prostatic hyperplasia. He was referred by the oncologist for comprehensive diagnostic testing involving fluorescence in situ hybridization (FISH), a BM biopsy for histopathological diagnosis, as well as flow cytometry and analysis of the aspirate due to a recent diagnosis of MM. FISH testing confirmed significant hyperploidy involving trisomy 9 and an extra copy of CCND1, with concomitant trisomy 11q confirming a t(11;14) translocation (Table 1).

**Table 1:** FISH findings The results showed significant hyperploidy involving trisomy 9 and an extra copy of CCND1 with concomitant trisomy 11q confirming a t(11;14) translocation FISH: fluorescence in situ hybridization; IgH: immunoglobulin heavy locus

FISH diagnosis			
Locus	Probe	Result	Cell counted
1p36.31/1q25.3	1p36/1q25	Normal	200
9q12	CEN9	Trisomy 9 (59%)	200
11q13.3/14q32.33	CCND1/IgH	Loss of IgH and extra copy of CCND1 (89%)	200
13q14.2/13q34	D13S319/13q34(G)	Monosomy 13 (88%)	200
14q32.33	IgH	Loss of IgH (94%)	200
17p13.1/17p11.1-q11.1	TP53/CEN17	Normal	200

The BM biopsy showed hypercellular marrow with 90% IgG/Kappa clonal plasmacytosis and no amyloidosis (Table 2). The BM aspirate diagnosis was similar to the biopsy, with 80% atypical plasma cells and orderly maturation (Table 3). Flow cytometry results showed IgG/Kappa monoclonal plasma cells with aberrant phenotype (Tables 4, 5, 6).

**Table 2:** Bone marrow histopathology findings The results showed the presence of hypercellular marrow with 90% IgG/kappa clonal plasmacytosis and no amyloidosis

Bone marrow histopathology diagnosis	
Specimen adequacy	Adequate
Cellularity	Moderately hypercellular (60%)
Marrow cellular composition	Marked diffuse plasmacytosis with diminished hematopoiesis
Myeloid maturation	Sparse but orderly
Erythroid maturation	Sparse but orderly
Myeloid/erythroid ratio	Normal (2:1)
Megakaryocytes	Moderately decreased with normal morphology
Iron stain	Moderately increased iron stores
Marrow reticulin	Normal
Special stains	PAS, Giemsa, iron, reticulin, Congo red, CD20, CD56, CD138, IgA, IgG, IgM,kappa, and lambda
Notes	The CD138+ plasma cells account for ~90% of the total marrow cellularity. They are IgG/kappa monoclonal with aberrant CD56 co-expression. No stromal amyloid deposits are noted by Congo red stain. Normal numbers of singly scattered CD20+ B-cells are present

**Table 3:** Bone marrow aspirate diagnosis The results showed similar to biopsy with 80% atypical plasma cells and orderly maturation

Bone marrow aspirate diagnosis		Number of cells counted: 200
Cell type	Result	Ref. range
Pronormoblasts	0.00%	0-1.0%
Baso. normoblasts	0.00%	0-5.0%
Poly. normoblasts	0.00%	6.0-16.0%
Ortho. normoblasts	5.50%	4.0-18.0%
Lymphocytes	6.50%	3.0-20.0%
Plasma cells	80.00%	1.0-4.0%
Promonocytes	0.00%	0-2.0%
monocytes	0.00%	1.0-4.0%
M:E ratio	2:01	2-4:1
Myeloblasts	1.00%	0-1.0%
Promyelocytes	0.00%	2.0-4.0%
Granulocytes	0.00%	5.0-19.0%
Eosinophils	0.00%	0.5-3.0%
Basophils	0.00%	0-1.0%
Metamyelocytes	2.50%	12.0-22.0%
Band	3.50%	8.0-16.0%
Segmented granulocytes	1.00%	7.0-22.0%
Segmented eosinophils	0.00%	0.5-4.0%
Segmented basophils	0.00%	0-1.0%

**Table 4:** Flow cytometry results - 1 The results showed IgG/kappa monoclonal plasma cells with aberrant phenotype (plasma cell markers)

Flow cytometry findings	
Gated population	Plasma cells 4%
CD38/CD138	99%
CD38/CD56	98%
CD38/CD19	90%
CD38/CD28	80%
CD38/CD81	87%
CD38/CD27	3%
CD38/CD117	91%
CD38/CD20	71%
CD38/kappa	98%
CD38/lambda	2%
CD38/IgA	3%
CD38/IgG	85%
CD38/IgM	1%

**Table 5:** Flow cytometry results - 2 The results showed IgG/kappa monoclonal plasma cells with aberrant phenotype (B-cell markers)

Flow cytometry	
Gated population	Lymphs 24%
CD19	2%
CD20	1%
CD38	<1%
CD19/kappa	61%
CD19/lambda	39%

**Table 6:** Flow cytometry results - 3 The results showed IgG/kappa monoclonal plasma cells with aberrant phenotype (T-cell markers)

Flow cytometry	
Gated population	Lymphs 24%
CD3	82%
CD4	45%
CD8	28%

The patient was informed about these findings, and he is presently being treated with Darzalex (daratumumab), a monoclonal antibody with affinity for the CD38 marker found on plasma cells. He is undergoing a four-cycle assessment and response protocol, with each cycle lasting two weeks. Following this, tumor reassessment is conducted through CT imaging, and the response is evaluated. The patient is currently on his fourth cycle. He has not experienced any adverse reactions to the medication and is tolerating it well. We will continue to monitor his progress and observe for any warning signs.

## Discussion

MM is the world’s second most common bone marrow malignancy [8]. This disease has an incidence of one out of 103 men and one out of 131 women, making it more likely to develop in males than in females [3]. It is found at the malignant end of a spectrum of many diseases known as plasma cell dyscrasias, representing a range of conditions marked by the proliferation of monoclonal plasma cells in the bone marrow. This proliferation leads to the production of monoclonal immunoglobulins, which in turn cause damage to vital organs such as the kidneys and bones [9]. Damage to these organs is the cause of key laboratory findings in MM, such as hypercalcemia and anemia.

In individuals with MM, the bone marrow environment exhibits elevated levels of interleukins and cytokines, playing key roles in both inflammatory and anti-inflammatory processes [10]. A key player in these processes is IL-1, a pro-inflammatory cytokine found in myeloid precursor cells in the blood. IL-1 further expresses two cytokines: IL-1 alpha and IL-1 beta. The latter is exclusively expressed in plasma cells in patients with MM and has two roles in the disease pathogenesis. It secretes IL-6, another pro-inflammatory cytokine that stimulates survival in myeloma cells, and it stimulates osteoclasts for bone resorption [11,12]. It is believed to be one of the major causes of hypercalcemia seen in patients with MM. Damage to the kidneys occurs as a result of cast nephropathy. An abundance of monoclonal light chains leads to a buildup in the tubules, forming aggregates and casts that consequently lead to inflammation and destruction of the kidneys [13].

While there are many mutations associated with MM, the five most common IgH mutations are t(11;14), t(4;14), t(14,16), t(14,20), and t(6;14). Of these, t(11;14) is found in approximately 16% of MM patients and is the most common variant [14]. This translocation involves CCND1 and IgH genes and exhibits a unique biological feature compared to other MM cell types, characterized by an increase in the presence of the B-cell lineage membrane protein CD20, higher levels of the B-cell receptor CD79a, and a decrease in the expression of both CD38 and CD56 [6,7]. CCND1, a gene involved in cell cycle regulation, is found overexpressed in 40% of MM patients. Some studies have shown that overexpression of CCND1 is associated with the worst prognosis of the disease, while others have found that it is tied to better outcomes. Hence, the prognostic significance of this gene dysregulation remains controversial [15]. IgH is known to be affected during various processes such as VDJ rearrangement and RAG complex-mediated variables such as those seen in mantle cell lymphoma t(11;14). However, in MM patients, the main mechanism of translocation occurs during class switching and recombination [16].

CD38 has emerged as an important therapeutic target for MM. It is involved in the regulation of calcium signaling and immune cell activation, proliferation, and migration [17]. CD38 is a transmembrane glycoprotein expressed at elevated levels in most MM patients, except those who carry the t(11;14) translocation. Studies have shown that patients with this mutation seem to carry significantly lower levels of CD38 expression, presenting challenges in treatment with anti-CD38 monoclonal antibodies such as daratumumab [18]. Daratumumab, an immunoglobulin G1 kappa monoclonal antibody, received FDA approval for the treatment of MM in 2015 [19]. It functions by stimulating antibody-dependent cell-mediated cytotoxicity (ADCC) in CD38-expressing cells. It is important to note that it does not induce ADCC in CD38-negative cells, making it highly specific [20].

## Conclusions

We discussed the case of a 75-year-old male who was referred to the clinic by his oncologist for comprehensive diagnostic testing. Upon review of the patient's test results, it was evident that he possessed a t(11;14) translocation, which is common in 15-20% of patients with MM, and showed a rare elevation in his CD38 marker levels. The patient is currently being treated with daratumumab on a four-cycle assessment and response protocol, followed by tumor reassessment using CT imaging and response evaluation. The patient is on his fourth cycle and has yet to show any adverse effects to the treatment, and is tolerating it well. To the best of our knowledge, this is the first report of a case with MM t(11;14) subtype with elevated CD38 marker levels. We believe our findings will be beneficial to the scientific community.
